# Artificial Intelligence in Heart Failure with Preserved Ejection Fraction

**DOI:** 10.3390/diagnostics16111597

**Published:** 2026-05-23

**Authors:** Xinyi Li, Chunyan Xu, Wenhui Deng, Yanting Zhang, Cong Liu, Lang Gao, Mengmeng Ji, Qing He, Zhenni Wu, Shuxuan Qin, Yixia Lin, Yuman Li

**Affiliations:** 1 Department of Ultrasound Medicine, Union Hospital, Tongji Medical College, Huazhong University of Science and Technology, 1277 Jiefang Avenue, Wuhan 430022, China; 13886177210@163.com (X.L.); xucy@hust.edu.cn (C.X.); wenhui0608@163.com (W.D.); zhangytcw@163.com (Y.Z.); liuc@hust.edu.cn (C.L.); m202176058@alumni.hust.edu.cn (L.G.); jimengmeng97@163.com (M.J.); hqingedu@163.com (Q.H.); babala0806@163.com (Z.W.); qsxqsx020508@163.com (S.Q.); linyixia@hust.edu.cn (Y.L.); 2 Clinical Research Center for Medical Imaging in Hubei Province, Wuhan 430022, China; 3 Hubei Province Key Laboratory of Molecular Imaging, Wuhan 430022, China; 4 Key Laboratory of Biological Targeted Therapy, Huazhong University of Science and Technology, Ministry of Education, Wuhan 430022, China

**Keywords:** heart failure with preserved ejection fraction, machine learning, artificial intelligence, diagnosis, deep learning

## Abstract

**Background:** Heart failure with preserved ejection fraction (HFpEF) is a heterogeneous syndrome characterized by frequent underdiagnosis, diverse etiologies, and limited therapeutic options. Given its complexity, artificial intelligence (AI) and machine learning (ML) offer promising avenues to decode high-dimensional, multi-modal healthcare data. This review aims to synthesize the current landscape of AI/ML applications in HFpEF, evaluating their potential to address critical unmet clinical needs. **Methods:** We conducted a comprehensive review of the literature focusing on AI/ML paradigms in HFpEF. Key methodological frameworks were examined, including supervised, unsupervised, semi-supervised, and reinforcement learning, alongside advanced techniques such as deep learning and natural language processing (NLP). The analysis focused on the application of these techniques across four domains: diagnosis, sub-phenotyping, risk prediction, and optimization of diagnostic modalities, with specific emphasis on studies incorporating external validation. **Results:** Current evidence demonstrates that AI approaches effectively enhance diagnostic accuracy and facilitate the identification of distinct HFpEF phenotypes beyond traditional classifications. These technologies show significant utility in refining prognostic assessments and optimizing diagnostic testing strategies. Furthermore, ML-driven analytics provide a robust framework for improving patient selection and streamlining clinical trial design, potentially overcoming historical barriers to drug development in this population. **Conclusions:** AI represents a transformative tool capable of dissecting the heterogeneity of HFpEF to enable precision medicine. While the potential to improve clinical outcomes is substantial, challenges regarding model interpretability, bias, and clinical integration persist. Future efforts must focus on rigorous external validation and prospective trials to ensure the responsible translation of these technologies into routine clinical practice.

## 1. Introduction

Heart failure (HF) is a common and complex disease impacting nearly 56.2 million individuals worldwide, with an increasing prevalence [1], and affecting approximately one in four adults in their lifetime [2]. By 2030, 8 million people in the United States will have HF and the associated costs will exceed 70 billion dollars [3]. Heart failure with preserved ejection fraction (HFpEF) constitutes nearly half of all HF cases and affects over 3 million adults in the United States alone [4]. This complex, heterogeneous syndrome poses significant challenges to clinicians and researchers due to its multifactorial etiology, underdiagnosis, and limited therapeutic options despite numerous clinical trials. Early diagnosis and timely clinical intervention are critical for mitigating disease progression and reducing the individual and societal burden of HFpEF [5,6,7]. Therefore, the inherent complexity and heterogeneity of HFpEF make it an ideal candidate for the application of artificial intelligence (AI) and machine learning (ML) approaches, which have shown great promise in navigating the high-dimensional, multi-modal data landscapes characteristic of modern healthcare [8].

Artificial intelligence, broadly defined as computer systems designed to perform tasks requiring human intelligence, encompasses various subfields including machine learning [9]. Machine learning focuses on developing algorithms that learn from data, identify patterns, and make predictions without explicit programming. Given the profound unmet clinical needs in HFpEF including its high underdiagnosis rate, marked phenotypic heterogeneity, limited therapeutic options, and suboptimal risk stratification, AI meothds have emerged as transformative tools with the potential to resolve longstanding bottlenecks in HFpEF management. This review therefore aims to systematically outline the current landscape of AI applications in HFpEF and discuss its associated challenges and opportunities.

## 2. Methods 

To comprehensively and systematically identify and integrate existing evidence in this field, we formulated the following literature search and screening strategy.

Literature Search: To obtain the most relevant academic literature, we searched several internationally renowned biomedical and engineering databases, including PubMed and Google Scholar. The search timeframe was set from January 2010 to May 2025 to cover the major advancements in this field over the past fifteen years. The search strategy employed a combination of keywords, integrating core terms describing the disease with terminology related to AI technology. The main keywords included: ”heart failure with preserved ejection fraction”, “HFpEF”, “HFpEF diagnosis”, “HFpEF phenotyping”, “diastolic dysfunction”, “artificial intelligence”, “machine learning”, “deep learning”, “convolutional neural network”, “natural language processing”, “supervised learning”, “unsupervised learning”, “phenomapping”, “clustering”, “risk prediction”. According to the characteristics of each database, we correspondingly used medical subject headings and their corresponding free-text terms, and constructed search queries using Boolean operators (“AND”, “OR”) to ensure the sensitivity and specificity of the search. 

For the initially retrieved literature, we first conducted a preliminary screening by reading titles and abstracts to exclude obviously irrelevant studies. For potentially eligible literature, we further obtained and evaluated the full text. Studies were included if they primarily focused on HFpEF and investigated the application of AI, machine learning, or deep learning models in its diagnosis, phenotyping, risk prediction, prognosis, treatment, or analysis of specific diagnostic modalities such as echocardiography, ECG and CMR. Eligible publication types were original research, systematic reviews, meta-analyses, and influential conference proceedings. We excluded editorials, comments, letters, case reports, and studies that did not apply AI methodologies or primarily focused on other heart failure types. 

As this article aims to provide a conceptual synthesis of existing knowledge rather than conduct a strict systematic review and meta-analysis, a formal record of the number of articles screened at each stage was not maintained. The final selection of literature was intended to provide a comprehensive, representative overview that illustrates key concepts, landmark studies, and emerging directions in the field, as described in the main text.

## 3. Overview of Machine Learning Techniques in Cardiovascular Medicine

### 3.1. Key Machine Learning Paradigms

Machine learning techniques relevant to HFpEF can be categorized into several key paradigms (Table 1), each with distinct applications:

Supervised Learning: This approach utilizes labeled training data, where each instance includes both input features (e.g., age, blood pressure, and echocardiographic parameters) and a corresponding output label (e.g., HFpEF diagnosis, mortality, or hospitalization). Supervised learning involves training models on labeled data by selecting, processing, and weighting input features in order to accurately predict the target outcome. When dealing with complex outcomes that require expert evaluation and annotation, researchers must label the source data. This can make the supervised learning approach more time-consuming and resource-intensive than unsupervised learning. Its goal is to construct a function that maps inputs to outputs, enabling accurate predictions on new and unseen data. Common supervised learning algorithms include logistic regression, support vector machines (SVMs) [10], and random forests [11], which have been applied to tasks such as predicting re-hospitalization and mortality in HFpEF patients [9]. SVMs have shown particular promise in lower-middle-income countries for predicting all-cause mortality in HF patients, achieving an area under the receiver operating characteristic curve (AUROC) of 0.77. This performance was demonstrated in a study by Mpanya et al., which highlights the utility of SVMs in resource-constrained settings [12].

Unsupervised Learning: In contrast, this paradigm operates on unlabeled data to discover intrinsic patterns and structures. This approach is particularly valuable for identifying disease mechanisms, genotypes, phenotypes or subgroups (sub-phenotypes). Clustering algorithms, such as k-means and hierarchical clustering, along with dimensionality reduction techniques like principal component analysis, have been instrumental in phenomapping efforts to classify HFpEF into more homogeneous subgroups with distinct clinical profiles and outcomes [9]. Hierarchical clustering is an algorithm that groups data based on similarity to another cluster by aggregating or dividing them as it moves up or down the hierarchy. This algorithm is often used to identify clusters of HFpEF patients when the number of clusters is not pre-defined. Conversely, partitioning algorithms are iterative methods that group the unlabeled dataset into different clusters. These algorithms are applied to identify clusters of HFpEF patients when the number of clusters to which patients will be assigned is pre-defined. Besides, principal component analysis is used for dimensionality reduction in ML. It converts observations of correlated features into linearly uncorrelated features via an orthogonal transformation. Principal component analysis is commonly employed to identify key features in ML algorithms. It has previously been used to identify key features of heart rate variability to assist in HF diagnosis. As Xie et al. highlight in their review, unsupervised learning has been crucial for phenomapping in HFpEF [13], with studies using various data elements and statistical techniques to identify unique subgroups since Shah et al.’s landmark 2015 study [14].

Semi-supervised Learning: This hybrid approach combines labeled and unlabeled data, which is advantageous when labeled data is limited, a common scenario in clinical research. Semi-supervised methods can enhance model performance by leveraging the larger volume of available unlabeled data while maintaining the guidance provided by the smaller labeled dataset [15]. It uses labeled data to guide the learning process and unlabeled data to capture more information and generalize more effectively. For example, Mpanya et al. [16] investigated the use of labeled and unlabeled patient data in a semi-supervised classification to identify HF patients. The study aimed to improve the class separation between HF and non-HF patients by leveraging additional unlabeled data. This study used a dataset containing patient records with labeled outcomes indicating the presence or absence of HF.

Reinforcement Learning: This paradigm focuses on developing algorithms that learn optimal sequential actions within a specific environment to maximize a defined reward. Although it has been applied less frequently in HFpEF research thus far, reinforcement learning has the potential to optimize treatment sequences and personalized therapeutic strategies [17].

### 3.2. Advanced Techniques: Deep Learning and Natural Language Processing

Deep Learning: As an extension of neural networks, deep learning excels at processing high-dimensional raw data such as images and signals. Convolutional neural networks (CNN) have shown promise in analyzing echocardiographic and cardiac MRI images. For instance, Unterhuber et al. [18] developed a CNN model that achieved an AUROC of 0.92 in distinguishing HFpEF patients from controls based on ECG data, with high sensitivity (0.98) and specificity (0.63). Recurrent neural networks (RNN), particularly long short-term memory networks, are well-suited for sequential data, such as vital signs and medical measurements over time. These networks can predict HF progression by analyzing temporal patterns in patient data [19]. A key advantage of deep learning is its ability to automatically perform feature engineering, reducing reliance on manual feature extraction by domain experts. The EchoGo Heart Failure v2 model, an updated 3D CNN developed for HFpEF detection using a single apical 4-chamber (A4C) transthoracic echocardiogram (TTE) video clip, exemplifies the practical application of deep learning in clinical cardiology, demonstrating robust performance across diverse patient populations [20]. Additionally, Chiou et al. [21] developed an AI-assisted system that provides fast (less than three minutes per case) and accurate prescreening for HFpEF based on apical 4-chamber view echocardiography. This system uses U-net, a conventional neural network with a U-shaped architecture consisting of contracting and expanding pathway focused on image features, to extract intrabeat dynamic changes (length, width, volume, and area data) in the left atrial and left ventricular (LV) [22]. These linear signals can used to train a 1-dimensional (1D) convolutional neural network for model prediction.

**Figure 1:**
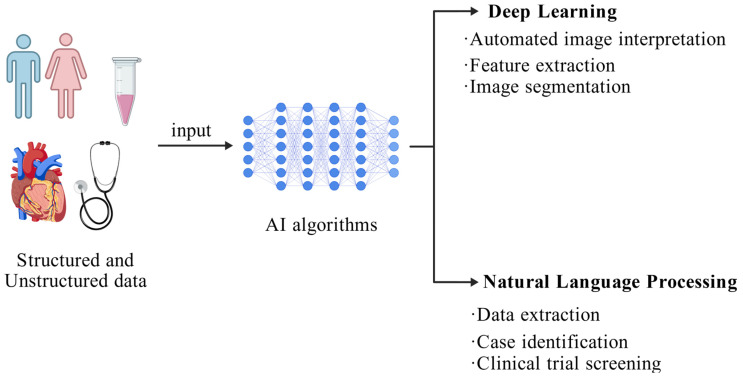
Overview of Deep Learning and Natural Language Processing.

Natural Language Processing (NLP): NLP enables the extraction of structured information from unstructured text data, such as clinical notes, discharge summaries, and imaging reports. with the development of transformer models like BERT (Bidirectional Encoder Representations from Transformers), this technology has become increasingly sophisticated and has been adapted for biomedical and clinical text analysis [23]. In HFpEF research, NLP facilitates efficient data extraction from electronic health records (EHRs) for various applications, including case identification and clinical trial screening [24]. In one study, the researchers have explored NLP for improving HFpEF detection and have demonstrated that undiagnosed HFpEF patients represent a high-mortality risk group identifiable through EHRs data analysis [25]. In another study, Marti-Castellote et al. [26] developed a Heart Failure NLP model that automatically adjudicates HF hospitalizations from medical records in 3 global clinical trials [27,28,29] and applied AI to extract evidence for individual HF symptoms, signs, and treatments. (These techniques mentioned above are summarized in Figure 1).

## 4. Applications of AI in HFpEF

### 4.1. Improved Diagnosis and Etiology Detection

A heart failure diagnosis depends on a patient’s medical history and physical examination, as well as imaging and laboratory data. However, routine imaging can be expensive. In addition, asymptomatic or mildly symptomatic HF may be easily overlooked if diagnosis relies on patient history alone. Late detection can result in a delay in the initiation of guideline-directed medical therapy, which can lead to preventable deaths or hospitalization. One of the most promising applications of AI in HFpEF is improving diagnosis and identifying underlying etiologies, particularly rare causes that are often underdiagnosed. Cardiac amyloidosis, for example, may account for 5–7% of HFpEF cases, yet it is frequently unrecognized despite the availability of novel therapies that can alter disease progression [30,31].

Several studies have demonstrated AI’s ability to detect such conditions using diverse data sources. For example, convolutional neural networks trained on echocardiographic images can accurately identify diseases such as hypertrophic cardiomyopathy, cardiac amyloidosis, and pulmonary arterial hypertension [32]. Deep learning models applied to ECG data can effectively detect structural heart diseases relevant to HFpEF [33]. Unterhuber et al.’s CNN model incorporates NT-proBNP values into its diagnostic algorithm, represents a landmark example of deep learning that can detect HFpEF in accordance with European Society of Cardiology (ESC) criteria. It has been successfully validated in an external cohort of at-risk individuals [18]. Sequential application of deep learning models—first to ECG data to identify high-risk individuals and then to echocardiographic data—has increased the positive predictive value for cardiac amyloidosis from 33% to 74–77% [34].

A recent external validation study of the EchoGo Heart Failure v2 model provides further evidence of the diagnostic utility of AI in HFpEF. In a case–control study involving 240 HFpEF patients and 256 age-, sex-, and echocardiogram-matched controls, the AI model demonstrated similar discriminative performance (AUROC: 0.798) to the H2FPEF score (AUROC: 0.788), with no statistically significant difference between the two groups. However, the AI model showed superior classification performance due to fewer intermediate classifications (15.1% vs. 61.7% for H2FPEF and 54.2% for HFA-PEFF). This addresses a critical limitation of traditional clinical scores, which often leave clinicians uncertain about further diagnostic steps [35]. Notably, the AI model’s continuous output provided significant incremental information beyond traditional scores, with a positive net reclassification index (NRI 0.40, 0.21–0.59) [35]. This suggests that AI can enhance diagnostic precision by reducing uncertainty, particularly in complex clinical cohorts with multiple comorbidities and conditions that mimic HFpEF—a scenario commonly encountered in real-world practice but often underrepresented in idealized research settings.

A collection of features obtained directly from heart sounds can be used as input for a ML algorithm to diagnose HF early. In one study, the researchers proposed a computer-assisted diagnosis system for chronic heart failure (CHF) based on cardiac reserve indexes analysis and heart sound characteristics. Using a dataset consisting of 88 healthy volunteers and 64 CHF patients, the system achieved a diagnostic accuracy of 95.39%, a sensitivity of 96.59%, and a specificity of 93.75%. This method outperforms back-propagation artificial neural networks and hidden Markov model, providing technical support for the point-of-care diagnosis of CHF [36]. However, it should be noted that this study is based on a relatively small cohort. Although the reported performance metrics are high, the small sample size may increase the risk of model overfitting and limit the generalizability of the results. In another study, Yang et al. [37] proposed a non-invasive method based on CNN and heart sounds for the early diagnosis of left ventricular diastolic dysfunction. To address the scarcity of samples in the heart sound database, they design a data augmentation method based on deep convolutional generative adversarial networks (DCGAN). The heart sound signals are preprocessed using an improved wavelet denoising method. Then, a logistic regression-based hidden semi-Markov model is used to segment the signals. These signals are then converted into spectrograms via short-time Fourier transform. Next, the proposed method is compared with six deep learning models, including VGG-16 and ResNet-18. The results show that the proposed CNN model achieves the optimal performance on the RS and DCGAN datasets, with an accuracy of 0.987, a sensitivity of 0.986, and a specificity of 0.988. These results confirm the effectiveness of heart sound analysis for early left ventricular diastolic dysfunction diagnosis and the value of DCGAN-based data augmentation. However, the reported high performance metrics urgently require prospective or external validation in independent clinical cohorts with larger sample sizes. This is necessary to confirm whether the model can maintain stable performance across broader and more heterogeneous populations, thereby evaluating its true clinical translation potential.

Additionally, several AI models have demonstrated promising results for HFpEF diagnosis. Chiou et al. developed an AI prescreening tool for HFpEF that uses the dynamic LV and LA changes within a cardiac cycle. This tool achieved high accuracy, sensitivity, and specificity in both internal and external validations [21]. One recent study developed a deep neural network that integrates multidimensional echocardiographic data. This network showed a higher AUROC than the 2016 ASE guidelines for predicting elevated LV filling pressure [38]. In another study, Chen and co-workers developed an AI-assisted system for LV diastolic function assessment. This system performed favorably compared to human experts. It can provide assessments using 2D strain metrics or single-view videos when Doppler variables are missing [39]. Unterhuber et al. utilized a CNN model to distinguish individuals with HFpEF from controls. Their study enrolled 1884 patients with exertional dyspnea and preserved ejection fraction (50%) in the derivation cohort and 203 volunteers from heart failure screening as the external validation cohort. The results showed that the AUROC of the model in the blinded test set of the derivation cohort was 0.92, demonstrating a high sensitivity and specificity [18].

### 4.2. Phenomapping and Sub-Phenotyping

The European Society of Cardiology Heart Failure Association (ESC HFA) has published a consensus document introducing the HFA-PEFF algorithm, a four-step diagnostic pathway designed to standardize the diagnosis of HFpEF [40]. The algorithm standardizes HFpEF diagnosis through a four-step pathway (P-E-F_1_-F_2_), utilizing echocardiographic and biomarker scores to stratify diagnostic certainty. Despite the advancement in diagnostic standardization, HFpEF remains a highly heterogeneous syndrome, characterized by both significant burden of comorbidities and pathophysiological abnormalities affecting multiple systems. The heterogeneity of HFpEF has hindered the development of effective therapies, as evidenced by numerous unsuccessful clinical trials. To bridge this gap, the ESC HFA has emphasized the utility of ML to decipher complex, multi-dimensional interactions within dense datasets. Beyond diagnosis, the HFA-PEFF framework aims to identify prevalent HFpEF phenotypes and propose evidence-based treatment strategies tailored to individual patient profiles. This tool not only identifies the most common HFpEF phenotypes but also formulates an evidence-based treatment approach for each phenotype, aiming to improve clinical treatment guidance [41]. By identifying HFpEF phenotypes via ML and other methodologies, the algorithm provides a framework for generating hypothesis and designing future clinical trials, which could guide the selection of personalized therapy. AI-driven phenomapping approaches have emerged as a means to identify more homogeneous subgroups (phenogroups) within the heterogeneous HFpEF population. Shah and colleagues pioneered this approach by applying unsupervised machine learning to dense phenotypic data. They identified three distinct HFpEF phenogroups with different clinical characteristics, and rates of cardiovascular hospitalization and death. These findings were replicated in an independent validation cohort, demonstrating the robustness of the approach [14]. Subsequent studies using similar techniques have identified overlapping phenotypes across different cohorts, suggesting that certain subgroups may represent distinct pathophysiological entities with differential therapeutic responses.

As detailed in the Xie et al.’s review, efforts to perform phenomapping in HFpEF have significantly increased using various data elements and statistical techniques [13]. Recent advances have extended phenomapping beyond structured clinical data to incorporate echocardiographic measurements and other modalities. For example, Segar et al. used unsupervised cluster analysis based on machine learning to analyze 61 mixed phenotypic variables from the Treatment of Preserved Cardiac Function Heart Failure with an Aldosterone Antagonist (TOPCAT) trial cohort in the Americas. This analysis led to the identification of three phenogroups of HFpEF with significant differences. This phenogroup classification was validated in both the non-echocardiographic TOPCAT cohort and the RELAX trial cohort, confirming that machine learning-based phenomapping can facilitate the development of individualized risk stratification and treatment regimen for HFpEF patients [42]. Chao and colleagues developed an unsupervised ML approach for diastolic function classification using nine diastolic function Doppler variables. They identified physiologically and prognostically distinct clusters [43]. Peters et al. reviewed phenomapping in HFpEF and noted insights, limitations, and future directions, including challenges with generalizability, single-center designs, and a lack of longitudinal data. These challenges have prevented widespread clinical implementation [44].

In recent years, unsupervised machine learning methods such as phenomapping and cluster analysis, have been increasingly applied in the field of cardiovascular diseases to uncover heterogeneity, guide precise stratification and precision medicine. Katz et al. [45] first conducted hypertension phenomapping and verified that unsupervised phenotypic clustering can identify clinically relevant hypertension subtypes, providing a basis for the targeted prevention of HFpEF. In another study [46], the researchers used automated hierarchical clustering to classify symptomatic HFpEF patients into abnormal and normal groups. A 2-year follow-up revealed that the normal group had a better prognosis than the abnormal group. The study concluded that isolated LV systolic reserve reduction is linked to HFpEF exercise intolerance and predicts a better prognosis than multiple abnormalities. Kaptein and colleagues analyzed 162 asymptomatic diastolic dysfunction patients via hierarchical clustering of 65 variables, identifying three phenotypic subgroups. The study found that diabetes mellitus, chronic kidney disease, atrial fibrillation, and diuretic use independently predicted HFpEF progression [47]. These findings support risk stratification and individualized interventions for these patients. Hedman et al. [48] employed a model-based machine learning clustering approach on 320 outpatients with stable HFpEF from the Karolinska-Rennes cohort, integrating 32 echocardiographic parameters and 11 clinical-laboratory indicators to identify six distinct phenogroups. These phenogroups differed significantly in terms of comorbidity prevalence, 15 plasma protein levels, and the composite endpoint of all-cause mortality or HF hospitalization. An Elastic Net model assigned new patients to phenogroups with a multiclass AUROC of 0.79, supporting HFpEF precise stratification, clinical trial design and the development of novel intervention strategies. While this study represents a more comprehensive phenotyping effort within a well-characterized cohort, the sample size remains moderate for deriving six complex phenogroups. While promising, indicates room for improvement in classification accuracy, and the clinical applicability of this specific stratification scheme awaits confirmation in external and prospective settings. Collectively, these studies demonstrate that data-driven clustering approaches can segment HFpEF populations into subtypes with potentially distinct pathological and prognostic profiles, suggesting new pathways for personalized management. However, most evidence to date originates from exploratory analyses in single-center or modestly sized cohorts. The clinical validity, stability, and actionable nature of the proposed subgroups require rigorous external validation in larger, diverse populations and assessment within prospective studies or trials to determine their ultimate value in guiding phenomapping.

Machine learning methods can improve HF prognostic assessment, optimize clinical phenotyping and treatment regimens, and guide HF clinical trial design [49]. Horiuchi et al. sought to identify clinically significant subgroups of acute heart failure (AHF) via non-hierarchical clustering of 77 variables in 345 consecutive AHF patients, using Cox regression for outcome analysis. Their findings indicate that cluster analysis can effectively distinguish clinical subtypes of AHF, providing a basis for personalized treatment and prognostic assessment [50]. Jones et al.’s study [51] aimed to distinguish the mechanistic differences between heart failure with reduced ejection fraction (HFrEF) and HFpEF. It employed a closed-loop model of the cardiovascular system, combined with TTE and right heart catheterization data from 31 patients (10 with HFrEF and 21 with HFpEF). The results of the study showed that HFrEF is driven by reduced left ventricular contractility, while HFpEF exhibits a heterogeneous phenotype. This study indicates that modeling the cardiovascular system and optimizing it with standard clinical data can classify HFpEF into distinct phenotypic subgroups, providing a basis for patient-specific treatment strategies. It should be noted that this is a proof-of-concept, mechanistic modeling study conducted in a small, carefully phenotyped cohort. While the approach is innovative for exploring pathophysiology, the derived subgroups and their clinical implications require validation in larger, independent patient populations to assess their robustness and generalizability. Using k-means clustering, Kobayashi and colleagues identified three echocardiographic phenotypes in the French STANISLAS cohort: including “mostly normal”, “diastolic changes (D)”, and “diastolic changes with structural remodeling (D/S)”. External validation in the Swedish Malmö Preventive Project cohort showed that the D phenotype (predominantly female, with elevated inflammatory biomarkers) and the D/S phenotype (predominantly male, with the highest left ventricular mass/volume and remodeling biomarkers) were linked to an increased risk of cardiovascular mortality or hospitalization for heart failure. These findings support the use of echocardiographic data-driven classification for HF risk stratification and early intervention in asymptomatic middle-aged individuals [52].

### 4.3. Molecular Mechanism of Action and Treatment

In order to figure out the exact mechanism of action of empagliflozin in HFpEF at the molecular level, Bayes-Genis et al. [53] used AI to model the molecular effects of empagliflozin in HFpEF. Eventhough HFpEF accounts for nearly half of all heart failure cases, there is no evidence-based treatment proven to improve patient outcomes [54,55]. Current therapies for HFrEF focus on modulating neurohormonal activation, but they have shown limited success in landmark HFpEF trials. Sodium-Glucose Co-Transporter 2 inhibitors (SGLT2i) are a new class of drugs being evaluated for use in HF. These drugs have demonstrated unexpected clinical benefits in HFrEF in trials, such as the DAPA-HF with dapagliflozin [56] and Emperor-Reduced with empagliflozin [57]. However, at the time of the study, pivotal trials investigating SGLT2i in HFpEF were ongoing, and the extra-renal cardioprotective mechanisms of SGLT2i (beyond glycemic control) remained unclear. Artificial Intelligence, particularly artificial neural networks (ANNs) and deep learning, is well-suited for dissecting complex biological networks [58]—an advantage the research team previously leveraged to study drug mechanisms in HFrEF [59]. To elucidate the cardioprotective mechanisms of SGLT2 inhibitors in HFpEF beyond glycemic control, the researchers utilized AI to systematically investigate the targets and pathways of empagliflozin. Bayes-Genis et al. [53] used AI models, including ANNs and deep learning, to predict that empagliflozin reversed 59% of protein alterations in HFpEF overall. Its therapeutic effects are mainly exerted by regulating oxidative stress in cardiomyocytes, and by influencing cardiomyocyte stiffness, myocardial extracellular matrix remodeling, concentric hypertrophy, and systemic inflammation, with a mechanism accuracy of 94%. Analysis of the AI mathematical models reveals a cascade of the molecular interactions and the key proteins most likely involved in empagliflozin’s therapeutic effects in HFpEF. Ongoing research provides solid molecular evidence for the drug’s clinical application and opens new avenues for utilizing AI technology to deeply understand drug mechanisms in complex diseases, thus advancing precision medicine.

HFpEF is a highly heterogeneous disease, which is considered the primary reason for the slow progress in developing effective treatments. This heterogeneity also explains the current lack of disease-specific therapeutic recommendations in clinical guidelines [60]. Although medications such as mineralocorticoid receptor antagonist can improve patient function, they have not demonstrated benefits in reduced morbidity or mortality. Subgroup analyses in traditional clinical trials often fail to capture true patient heterogeneity. Though capable of identifying groups with similar clinical characteristics, phenotypic clustering methods cannot predict therapeutic responses [61]. In contrast, outcome-oriented machine learning algorithms can model more complex variable interactions and account for individual heterogeneity. These algorithms have shown superior performance over traditional statistical models when analyzing complex data [62]. In a recent study, the author and colleagues applied cluster analysis to data from the AIdo-DHF trial and identified a subgroup (38% of patients) who responded to spironolactone treatment by improving their E/e’ ratio. Based on these findings, an XGBoost classifier was developed and validated in the TOPCAT cohort. Validation results confirmed that spironolactone significantly reduced the occurrence of the primary endpoint only among patients predicted as “responders” (35% of the cohort), with no significant benefit observed in those predicted as “non-responders” (65% of the cohort) [63]. In another study, Li et al. [64] addressed the core challenges of significant clinical heterogeneity and limited effective treatments in HFpEF. Using a two-stage unsupervised DeepCluster machine learning model, it identified three distinct phenotypes based on 107 clinical variables from 2147 HFpEF patients. The phenotyping demonstrated 96.0% consistency through internal leave-one-out cross-validation and was further validated in external cohorts including TOPCAT and UMHS. Significant differences in prognosis and treatment response were observed among the phenotypes. This work provides critical real-world evidence to inform personalized and precise treatment strategies for HFpEF. Besides, Zhou et al.’s study [65] based on data from the TOPCAT clinical trial, employed the causal forest algorithm to investigate the heterogeneous treatment effect (HTE) of spironolactone in HFpEF. The analysis identified three key stratification variables which were used to classify 3312 patients into four distinct subgroups. The results demonstrated that spironolactone significantly reduced the risk of the primary composite endpoint in the subgroup with high BMI and high WBC. Conversely, it increased the risk of adverse events in the subgroup with low BMI and low ALP. No significant heterogeneity in the risk of drug discontinuation was observed across the subgroups. This work provides a machine learning-based stratification framework to guide individualized, precision pharmacotherapy for HFpEF. These researches strongly demonstrate that machine learning-based models can distinguish HFpEF subgroups effectively, thereby breaking through the traditional all-or-none efficacy evaluation framework and providing key methodological support for shifting from population-based treatment to individualized treatment.

### 4.4. Risk Prediction and Prognostication

Accurate risk prediction is crucial for optimizing HFpEF management and resource allocation. Traditional risk models for HFpEF, such as the I-PRESERVE Score and ARIC Score, have demonstrated only modest performance [66]. AI offers the potential to develop more robust predictive models by leveraging diverse data sources and accounting for complex, non-linear relationships between variables. The Machine Learning Assessment of Risk and Early mortality in Heart Failure (MARKER-HF) risk model exemplifies this potential, using just eight laboratory variables to predict 90-day and 1-year mortality with performance comparable to that of more data-intensive models [67,68]. This parsimony facilitates integration into EHR systems, enhancing clinical utility. The prognostic value of AI models for HFpEF has been further validated in recent research. The EchoGo Heart Failure v2 model demonstrated strong associations with adverse outcomes: a positive AI diagnosis was associated with a 2.56-fold increased risk of the composite endpoint of death or HF hospitalization, a 2.54-fold increased risk of mortality, and a 3.15-fold increased risk of HF hospitalization. These associations were consistent across quartiles of the model’s continuous output, with the highest quartile demonstrating a 3.95-fold increased risk of the composite outcome compared to the lowest quartile. Such findings confirm that AI-derived diagnostic information correlates with long-term outcomes, thereby reinforcing its clinical relevance beyond mere diagnostic classification [35].

Machine learning models are demonstrating increasingly significant clinical value in the risk stratification, prognostic assessment and management of HFpEF. One study developed a supervised ML model using noncontrast CMR imaging to predict HF hospitalization in HFpEF patients, significantly outperforming a basic clinical model [69]. In another study [70], Naghavi and colleagues found that AI-enabled cardiac chamber volumetry in coronary artery calcium scans significantly outperformed NT-proBNP and the Agatston score in predicting incident HF over 15 years. Zheng et al. [71] utilized electronic health records from 746 Chinese HFpEF patients. They selected 12 key features by univariate analysis combined with the least absolute shrinkage and selection operator (LASSO) algorithms, and constructed machine learning models like XGBoost, random forest, neural network and logistic regression. The results showed that the XGBoost model can accurately predict the 90-day readmission risk of HFpEF patients, providing a basis for early clinical intervention. Additionally, the Shapley Additive exPlanations (SHAP) method identified the top 12 risk features, including activities of daily living, left atrial dimension, left ventricular end-diastolic diameter, etc. These studies collectively indicate that machine learning methods can construct highly effective risk prediction tools by integrating clinical data and image features. This not only improves the accuracy of predicting adverse events in HFpEF patients but also provides robust support for achieving individualized and proactive clinical management decisions.

Utilizing data ranging from circulating proteins and genomics to epigenetics, these studies demonstrate the significant potential of integrating multi-level biological information in prognosis assessment. Gao et al.’s study [72] developed an SVM model that incorporated circulating biomarkers, including endoglin—a marker of inflammation and endothelial dysfunction, to predict 2-year all-cause death in acute HFpEF patients. This study highlights the value of combining molecular markers with ML for enhanced risk stratification. Zhou and colleagues [73] demonstrated that the Genetic Algorithm–Kernel Partial Least Squares (GA-KPLS) model achieved high accuracy in predicting survival status in HFpEF patients utilizing gene expression data. This approach captures nonlinear relationships in genomic data and offers insights into the molecular underpinnings of prognosis. The HFmeRisk model, developed by Zhao et al., integrates DNA methylation profiles with clinical features using LASSO, XGBoost, and deepFM algorithms for early HFpEF risk assessment [74]. This represents a novel direction in incorporating epigenetic factors into predictive models and could enable risk stratification before clinical manifestation [75]. Li et al. [76] and Chen et al. [77] both reported that XGBoost outperformed other algorithms (LR, random forest, SVM) in predicting in-hospital mortality among HF patients in ICU settings, with SHAP methods enhancing interpretability by identifying key predictors. These studies not only confirm the advantages of machine learning in integrating complex multi-omics data with clinical features, but also highlight that future model development must focus on external validation, clinical operability, and cross-population applicability to advance their translation into real-world clinical practice.

### 4.5. AI Applications in Specific Diagnostic Modalities for HFpEF

#### 4.5.1. AI-Assisted Echocardiography

Echocardiography is a cornerstone in HF diagnosis, particularly important for HFpEF assessment which relies heavily on diastolic function evaluation. However, detailed echocardiography is resource-intensive, and its results are often obtained by visual estimation rather than precise calculation [78]. Considering this problem, widely-adopted commercial software has been developed for analyzing echocardiography data, such as EchoPAC by GE healthcare and QLAB by Philips, etc. [79]. These technologies address the operator-dependent variability in echocardiography. Zhu et al. [80] developed a deep residual CNN that achieves extremely high accuracy in classifying multiple echocardiographic views. For segmentation, Zhang et al.’s U-Net-based model [32] achieved intersection over union metrics of 0.72–0.90 for LV segmentation, while Zamzmi et al.’s Trilateral Attention Network [81] and Wang et al.’s generative AI approach [82] showed improved performance in cardiac region segmentation. Khamis and co-workers utilized a multi-stage classification algorithm that employed spatio-temporal feature extraction and supervised dictionary learning approaches to enhance the automatic recognition and classification accuracy of echocardiograms [83]. Tromp et al. [84] developed a fully automated deep learning workflow that reliably classified diastolic dysfunction. Their assessment of the E/e’ ratio demonstrated strong agreement with expert measurements. This addresses a key challenge in HFpEF diagnosis because assessment of LV filling pressure using echocardiography is complex and requires accurate measurement of multiple parameters.

AI provides new tools to enhance diagnostic objectivity and precision in heart diseases, particularly in HFpEF, by optimizing image quality assessment, enabling automated functional measurements, and interpreting complex myocardial motion patterns. One study examined the impact of two echocardiographic image input methods (the average of ten images and ten originally selected images) and a mislabeled-image database on the accuracy of LVEF prediction model. Using 17,000 labeled images covering five standard views from 340 patients and a convolutional neural network with 5-fold cross validation, the optimal model based on ten originally selected images achieved 98.1% view classification accuracy with only 1.9% mislabeling. The EF model maintained good accuracy with 1.9% mislabeled images, confirming that this CNN algorithm enables clinically feasible echocardiographic view classification [85]. Another study [86] developed and tested a fully automated machine learning algorithm (AutoEF) that estimates LVEF without the need for volume measurements. Trained on a database of over 50,000 echocardiographic studies, AutoEF was feasible in all 99 independent test patients and excellent agreement with three experts’ volume-based reference values. It showed high sensitivity and specificity for detecting LVEF ≤ 35%, matching clinical readers’ performance. Sanchez-Martinez et al. [87] proposed an unsupervised multiple kernel learning (MKL) method to characterize the myocardial motion patterns of patients with HFpEF. The results showed that multi-feature joint analysis could improve the phenotypic characterization of HFpEF. These studies collectively demonstrate the potential of AI in echocardiography analysis. By ensuring image reliability and enabling efficient automated quantification, these studies lay a solid technical foundation for intelligent diagnosis and show broad prospects for clinical translation. (The technologies mentioned above are summarized in Table 2).

#### 4.5.2. AI-Assisted ECG Analysis

ECG is widely performed with little cost and modern machine learning models can identify different wave morphologies (e.g., QRS complexes, P and T waves) with high precision. Deep learning convolutional neural networks have been adapted to analyze the routine 12-lead ECG, resulting in AI models that mimic human interpretation of ECG. These models have the potential to provide greater diagnostic fidelity and workflow efficiency than traditional rule-based computer interpretations [88]. For example, Unterhuber et al. [18] developed a CNN model based on 12-lead ECG to detect HFpEF. The model achieved high sensitivity in derivation cohort and the external validation cohort. Cho et al.’s CNN model [89] for detecting HFrEF achieved high AUROC in internal and external validation, respectively. Features such as lateral/anterior wall leads, heart rate, QT interval, QRS duration, and T-axis showed high correlation. Sangha et al.’s CNN approach [90] for screening LV systolic dysfunction using 12-lead ECG images performed well in the internal and external validation cohorts for detecting LVEF < 40%. Kalmady et al.’s ResNet-based DL model [91] outperformed XGBoost in predicting multiple cardiovascular conditions, including HF, with ~5% higher AUROC for 8 conditions. Lee and colleagues [92] developed an AI-enabled ECG model to identify diastolic dysfunction and increased filling pressure determined by echocardiography. The model performed well in detecting increased filling pressure.

Integrating AI with ECG analysis technologies provides new perspectives for early HFpEF identification and gender-specific manifestations study, allowing conventional electrocardiograms to demonstrate greater clinical value in disease screening and risk stratification. In a recent study [93], the authors applied machine learning approaches to compare gender-specific ECG parameters in patients with HFpEF. Among these methods, the random forest model achieved an average accuracy of 84.7%. Kwon et al.’s study [94] developed and validated an interpretable deep learning model based on an ensemble neural network that enables the early detection of HFpEF using 12-lead, 6-lead, and single-lead ECG. This model provides an economical and convenient screening tool for HFpEF that can be used in combination with conventional or wearable ECG devices to facilitate early disease intervention. These studies further indicate that AI-based ECG analysis not only enables early screening and gender-difference analysis of HFpEF but also offers a basis for individualized risk assessment, facilitating the transformation of electrocardiography from a traditional diagnostic tool into an intelligent, precise disease management platforms. (These applications are summarized in Table 3).

#### 4.5.3. AI-Assisted Cardiac Magnetic Resonance (CMR)

CMR provides detailed structural and functional information for assessing HFpEF. AI can definitely improve the efficiency and accuracy of CMR in HFpEF assessment by automating complex image processing and enhancing accuracy. Xie et al. [95] developed a four-dimensional self-supervised learning framework for HF classification using cine CMR. This framework shows the potential to improve diagnostic accuracy and assist physicians in choosing personalized treatments. Additionally, Lehmann et al. [96] developed a non-invasive method using AI-enhanced CMR to predict the diagnostic results and left ventricular end-diastolic pressure in patients undergoing CMR. The study indicates that these AI models can easily be integrated into clinical practice, adding value to the CMR information and facilitating disease classification and diastolic function prediction. Wang et al. [97] developed and validated a deep learning-based, two-stage CMR interpretation paradigm for screening and diagnosing 11 types of cardiovascular diseases. Both the screening model and the diagnostic models demonstrated excellent performance on internal and external datasets. Furthermore, the diagnostic model outperformed cardiologists in diagnosing pulmonary arterial hypertension and could identify CMR features that are difficult for humans to detect. This research provides key technical support for improving the efficiency of CMR interpretation and promoting the widespread adoption of cardiovascular disease screening and diagnosis. These studies demonstrate that by integrating information from cardiac magnetic resonance imaging with deep learning technologies, AI can not only enhance the efficiency of cardiovascular diseases screening and diagnosis but also assist physicians in making precise functional assessments and classification, providing an efficient, reliable, and insightful intelligent tool for clinical decision-making. (The applications mentioned above are summarized in Table 4).

#### 4.5.4. Wearable Devices and Remote Monitoring

Wearable technologies incorporating AI models can offer rapid diagnoses for patients and consumers. Although many models were developed using 12-lead ECG data, some studies have proved that these models perform well with single-lead ECGs as well [98]. In one recent study [99], the researchers developed a noise-adapted AI model that can detect left ventricular systolic dysfunction (LVSD) in noisy single-lead ECGs obtained from portable and wearable devices. The noise-adapted model demonstrated excellent performance, significantly outperforming the standard model. Attia et al.’s prospective study [100] validated the feasibility of detecting LVSD by using single-lead ECGs from Apple Watches combined with an AI algorithm. Inan et al.’s ML-assisted wearable technology distinguished compensated and decompensated HF states using seismocardiogram signals, showing potential for remote monitoring of HFpEF patients [13].

Currently, non-invasive remote monitoring has not yet been shown to reduce hospitalization rates in randomized controlled trials, primarily due to the indirect (non-linear) relationship between the measured biological signals and patients’ congestion status. In contrast, there is growing evidence supporting the direct monitoring of intracardiac and pulmonary artery pressure. Among the available solutions, CardioMEMS, a direct pulmonary artery pressure monitoring system, was validated in the large-scale randomized CHAMPION trial. The system demonstrated excellent safety, with an extremely low rate of device-related complications and no failures of the pressure sensor. However, CardioMEMS faces challenges in real-world applications, including selection bias in study populations. The trial population differs from the general HF population: the average age of patients in the trial was 61 years, compared to over 70 years in real-world settings. There were also differences in the distribution of ejection fractions. Furthermore, left atrial pressure monitoring was not supported because the LAPTOP-HF trial investigating this approach was terminated early due to excessive implant-related adverse events [101]. (These applications are summarized in Table 5).

## 5. Challenges and Pitfalls

### 5.1. Data Quality and Bias

AI performance hinges on the data quality, yet clinical datasets suffer from several flaws, especially compounded by inherent HFpEF’s specific diagnostic ambiguities that fundamentally compromise the authenticity and consistency of training labels. Notably, HFpEF lacks a universally recognized gold-standard diagnostic benchmark, with persistent diagnostic discordance between invasive hemodynamic evaluation and non-invasive scoring criteria including H2FPEF and HFA-PEFF. First is incompleteness. EHRs often lack key variables. For example, 25% of patients in EchoGo’s validation dataset had missing NT-proBNP, which biased models toward variables like age and blood pressure [35]. Secondly, selection bias manifests in multiple dimensions. For example, training data overrepresents hospitalized patients and underrepresents community-dwelling HFpEF cases with milder symptoms. More broadly, such data are mostly derived from specific populations in selected medical centers. These populations tend to include patients with typical symptoms and few comorbidities, while neglecting HFpEF patients who are elderly, women, ethnic minorities, or have complex underlying conditions, such as diabetes or chronic kidney disease. Given the extremely high clinical and phenotypic heterogeneity of HFpEF itself, this dual-layered selection bias limits models’ ability to detect early-stage disease and cause the features learned by the model to fail to represent the highly heterogeneous patient group in the real world. This leads to misdiagnosis or missed diagnosis in application. Additionally, demographic underrepresentation is another critical aspect of this bias. EchoGo’s cohort included fewer Black patients in the control group (6.6%) than in the case group (18.3%), resulting in lower accuracy in Black populations. Such disparities perpetuate healthcare inequities [35].

Beyond issues of selection and completeness, the lack of data quality and standardization further undermines the reliability of clinical datasets. This issue is particularly prominent in HFpEF research, as its diagnosis is highly dependent on unified echocardiographic parameters, biomarker detection and clinical evaluation criteria. Different medical institutions use different models of detection equipment (e.g., echocardiography and ECG machines), and there are differences in operational specifications and parameter settings for data collection. This results in inconsistent manifestations of the same indicator across different datasets. Additionally, there is no unified standard for data storage formats, and clinical texts (e.g., medical records and examination reports) contain irregular expressions and incomplete information. These problems make data preprocessing and integration more difficult, which directly affects the stability and accuracy of model training.

Additionally, restrictions on data privacy and sharing pose a significant obstacle. Medical data involve patient privacy, and strict privacy protection regulations limit the sharing of data across institutions and regions. Most studies can only develop models based on small-scale, single-center datasets. This restricts the accumulation of sufficiently large and diverse data sets, meaning large medical data sets are rarely available and model performance improvement is limited. However, access to big data is essential for developing AI diagnostic models, especially for capturing the full spectrum of heterogeneous HFpEF subtypes and comorbidity profiles. Lastly, in real-world hospital setting, different medical data are often stored across multiple servers and, in some cases, in paper records. Even if AI produces highly accurate prognostic models, their effectiveness could be limited if hundreds of prediction parameters are scattered across various systems and must be entered manually.

### 5.2. Generalizability and Interpretability

Models perform poorly in external validation due to “dataset shift”, which refers to the differences between the training and real-world populations. For HFpEF research, this dataset shift is further amplified by disease-specific diagnostic heterogeneity, including inconsistent adoption of LVEF cut-off values (LVEF ≥ 50% for HFpEF vs. ≥40% for HFmrEF) across cohorts, diagnostic drift induced by the 2021 ESC and 2022 AHA/ACC/HFSA guideline updates, and persistent discordance between invasive hemodynamic criteria and non-invasive H2FPEF/HFA-PEFF scoring systems. This essentially reflects the insufficient generalizability of current AI models, most of which are mostly trained and validated using single-center datasets and lack multi-center, large-sample external validation processes. For example, the AUROC for EchoGo v2 decreased from 0.95 in internal validation to 0.798 in a diverse cohort, partly due to variations in echocardiogram quality and patient comorbidities. Single-center studies exacerbate this issue. Seventy percent of phenomapping studies use data from less than three centers, which limits their applicability across healthcare systems [35]. Differences in patient characteristics, diagnosis and treatment habits, and data distribution across different medical environments further lead to decreased model performance in new clinical scenarios, making it difficult to apply them widely in hospitals of varying levels or regions. Additionally, biases in AI algorithms often arise from nonrepresentative datasets, leading to biased predictions or outcomes in new populations. Overfitting is another common issue that causes the poor generalizability of AI models. Notably, HFpEF exhibits extremely high phenotypic and clinical heterogeneity with diverse clinical subtypes and complex comorbidity profiles, making AI models particularly prone to overfitting to localized single-center population features. An overfit model performs well on the training data but poorly on validation or test data sets. Several studies have demonstrated the poor generalizability of HF scoring systems in new populations. For instance, the famous Framingham Risk Score could overestimate or underestimate risk in non-US populations [102,103].

In addition to generalizability issues, AI models used in clinical practice face challenges related to poor interpretability and lack of robustness. Complex algorithms, such as deep learning, have a “black box” characteristic in their decision-making processes, which makes them difficult to interpret. In the context of HFpEF, such black-box models fail to explicitly clarify how echocardiographic indices, circulating biomarkers, and clinical comorbidities jointly contribute to diagnostic judgments, nor can they align model decisions with established HFpEF pathophysiological mechanisms. Moreover, the absence of a universal gold-standard diagnostic reference for HFpEF further complicates the interpretation of black box AI outputs. These models cannot clearly demonstrate which clinical features and physiological mechanisms they rely on to make diagnostic or prognostic judgments. This makes it difficult for clinicians to understand the basis of the model’s conclusions. They are unable to verify their rationality or explain them to patients, which affects the acceptance and trust of the model in clinical practice. Regarding robustness, models are sensitive to noise and outliers in data. For example, ECG data collected by wearable devices may contain noise due to motion or environmental interference, and there may be entry errors in clinical data, all of which can cause deviations in model decisions. This issue is more prominent in HFpEF cohorts, where elderly patients and those with multiple chronic conditions often have incomplete or noisy clinical records. Additionally, models have a weak ability to identify rare etiologies (e.g., cardiac amyloidosis) because such cases account for an extremely low proportion in the training data, making it difficult for the model to learn their typical features. This greatly limits the ability of AI tools to differentiate rare HFpEF subtypes from ordinary idiopathic HFpEF in clinical practice.

### 5.3. Clinical Application

Most AI tools are developed independently and lack standardized interfaces with electronic health record systems and hospital information systems. The lack of unified docking standards is particularly prominent for specialized AI tools targeting HFpEF diagnosis and subtype identification. This forces clinicians to switch between different systems, which adds to their workflow and reduces their willingness to use the tools. Additionally, the format of AI model output results does not align with the requirements for clinical diagnosis and treatment reports. This necessitates manual secondary collation, which impacts application efficiency. Furthermore, there are no unified guidelines or norms for applying AI to HFpEF diagnosis and treatment, including no clear consensus on the applicable population of AI tools, optimal application timing in the HFpEF diagnostic workflow, and standardized criteria for interpreting AI results combined with H2FPEF/HFA-PEFF scores and invasive hemodynamic indicators. Without a basis, clinicians find it difficult to judge the reliability of model results and establish standardized diagnostic and treatment pathways. Clinicians are reluctant to act on AI outputs they cannot explain, particularly for high-stakes decisions such as amyloidosis diagnosis. Therefore, AI-generated results should be carefully interpreted within the framework of medical knowledge. At the same time, some clinicians have insufficient understanding of the principles and application value of AI technology, holding a resistant attitude and worrying that model results will replace their own judgments. Meanwhile, operating AI tools and interpreting results require certain computer and data analysis skills that exist medical personnel lack, which limit the promotion and use of AI tools. Moreover, HFpEF patients often have multiple underlying diseases (e.g., hypertension, diabetes, chronic kidney disease, and atrial fibrillation). These comorbidities do not exist independently but interact synergistically to disrupt multi-system homeostasis, jointly driving HFpEF disease progression and long-term prognosis. Most existing models include comorbidities as ordinary features, yet they fail to fully consider the interaction between comorbidities and their complex impact on HFpEF, leading to reduced accuracy in the models’ predictive results.

## 6. Future Directions

The optimization of AI application in HFpEF should be promoted from multiple dimensions. At the data level, cross-regional and multi-center dedicated databases are being established, and unified data standards are being formulated. Technologies such as federated learning are being used to enable data sharing under privacy protection. Meanwhile, multi-modal feature systems are constructed by integrating imaging, molecular, textual, and real-time data from wearable devices. Rare case data are also accumulated through rare etiology registration systems. For instance, integrating CMR T1 mapping (myocardial fibrosis), RNA sequencing (inflammatory pathways), and smartwatch activity data could help identify subgroups at high risk for exercise-induced decompensation. Platforms like the GENERATOR HF DataMart, which aggregates real-world data, will facilitate such integration [104]. At the technical level, explainable AI is developed using methods like SHAP and LIME (Local Interpretable Model-agnostic Explanations) to generate intuitive reports and enhance clinical trust. Dynamic models are built with longitudinal data for real-time disease early warning. Unsupervised and supervised learning are combined to optimize HFpEF subtype classification and support personalized treatment. Technologies such as generative adversarial networks are adopted to improve model robustness. Fairness-related features are integrated to avoid discriminatory biases. At the clinical level, guidelines for AI application must be formulated to clarify usage specifications, develop standardized interfaces for seamless connection between AI and existing medical systems, conduct large-scale multi-center clinical trials to verify the value of AI tools, and strengthen AI knowledge training for medical staff. In terms of application scenarios, AI can be used for the early screening and risk prediction of HFpEF, as well as for assisting in the development of new drugs and precision treatments. Additionally, it can be used to construct remote monitoring platforms combined with wearable devices for full-cycle disease management.

## 7. Conclusions

In conclusion, AI has the potential to transform the treatment of HFpEF by addressing diagnostic ambiguity, heterogeneity, and poor risk prediction. AI technologies, such as machine learning algorithms, deep learning models, have shown promise in improving the accuracy and efficiency of HFpEF diagnoses. Additionally, AI can identify patterns and correlations that may be overlooked by traditional diagnostic methods, leading to early detection and personalized treatment for different patients. However, challenges such as generalizability, bias, and interpretability must be addressed. With rigorous validation and equitable implementation, AI can advance precision medicine in HFpEF and improve patient outcomes for this recalcitrant syndrome.

## Figures and Tables

**Table 1:** Four Machine Learning Paradigms in HFpEF Research.

Machine Learning Paradigm	Core Data Type	Function	Common Algorithms	Application Cases/Value in HFpEF Research	Ref.
Supervised Learning	Labeled training data (each instance comprises input features and a corresponding output label)	Learn an input-to-output mapping function to enable accurate predictions on new, unseen data	Logistic Regression Support Vector Machines (SVM) Random Forests	1. Predicting re-hospitalization and mortality in patients with HFpEF 2. Predicting all-cause mortality in HF patients in low- and middle-income countries (SVM models achieved an AUROC of 0.77, making them well-suited for resource-constrained settings)	[9,10,11,12]
Unsupervised Learning	Unlabeled data	Uncover intrinsic patterns and structures in data, with a focus on identifying subgroups (sub-phenotypes) within heterogeneous populations	Clustering algorithms (k-means, Hierarchical Clustering) Dimensionality reduction techniques (Principal Component Analysis, PCA)	1. Supporting phenomapping efforts for HFpEF 2. Stratifying the heterogeneous HFpEF population into homogeneous subgroups characterized by distinct clinical profiles and outcomes	[9,13,14]
Semi-supervised Learning	Mixed data (a small subset of labeled data paired with a large volume of unlabeled data)	Leverage guidance from limited labeled data while utilizing extensive unlabeled data to enhance model performance	Centered on the core logic of “mixed data utilization”	Addressing the common challenge of limited labeled data in clinical research and strengthening model generalization	[15,16]
Reinforcement Learning	Data tailored to “environment-action-reward” interaction scenarios; no explicit “label” framework	Develop algorithms that learn optimal sequential actions within a specific environment to maximize a predefined reward	Focused on the core logic of “sequential optimization”	Currently underutilized in HFpEF research but holds promise for optimizing treatment sequences and personalized therapeutic strategies for HFpEF patients	[17]

**Table 2:** Echocardiography in HF Diagnosis and Related Technological Advances.

Core Technology	Validation Type	Key Content	Technology/Method	Specific Achievements/Performance Metrics	Ref.
Echocardiographic View Classification Technology	Internal	Addresses the issue of “operator-dependent variability” in echocardiography; enables automatic classification of multiple views	Deep Residual Convolutional Neural Network (Deep Residual CNN)	Accuracy of 99.1% for classifying multiple echocardiographic views	[80]
Cardiac Structure Segmentation Technology	Internal	Improves segmentation accuracy of cardiac regions (especially the left ventricle) and reduces human error	1. U-Net model (for LV segmentation) 2. Trilateral Attention Network 3. Generative AI approach	1. Intersection over Union (IoU) for LV segmentation: 0.72–0.90 2. Improved performance in cardiac region segmentation (no specific values mentioned)	[32]
Automatic Recognition and Classification Technology for Echocardiograms	Internal	Enhances the accuracy of automatic recognition and classification of echocardiograms via a multi-stage algorithm	Multi-stage classification algorithm (integrating spatiotemporal feature extraction [Cuboid Detector] and supervised dictionary learning [LC-KSVD])	1. Diagnostic accuracy for A2C images: 97% 2. Diagnostic accuracy for A4C images: 91% 3. Diagnostic accuracy for ALX images: 97% 4. Average recognition rate: 95%	[81,82,83]
Automatic Classification Technology for Diastolic Dysfunction	External and Prospective	Addresses a key challenge in HFpEF diagnosis (echocardiographic assessment of LV filling pressure is complex and requires accurate measurement of multiple parameters); enables automatic classification of diastolic dysfunction	Fully automated Deep Learning (DL) workflow	1. Reliably classifies diastolic dysfunction 2. Good agreement between E/e’ ratio measurements and expert-derived values	[84]
Automatic LVEF Estimation Technology (AutoEF)	Internal	Developed a fully automated machine learning algorithm (AutoEF) that estimates LVEF without volume measurements, mimicking human experts by assessing ventricular contraction degree. Trained on over 50,000 echocardiographic studies.	Fully automated machine learning algorithm (AutoEF)	1. In testing (99 independent patients): feasible in all patients, mean absolute deviation of 2.9% 2. For LVEF ≤ 35% detection: sensitivity = 0.90, specificity = 0.92 3. Performance comparable to clinical readers (r = 0.94, bias = 1.4%, limits of agreement = ±13.4%); de-trending correction further improved accuracy.	[86]
Myocardial Motion Pattern Characterization Technology for HFpEF (Unsupervised MKL Method)	Internal	Proposed an unsupervised multiple kernel learning (MKL) method to characterize myocardial motion patterns in HFpEF patients, combining multiple sets of temporally aligned myocardial velocity traces (resting and exercise states) and cardiac event temporal information.	Unsupervised multiple kernel learning (MKL) method	1. Enrolled 55 subjects (22 healthy, 19 HFpEF, 14 breathless); improved HFpEF characterization via joint feature analysis 2. Diastolic features in exercise state and temporal deformation features contributed significantly to diagnosis 3. Distinguished different HFpEF pathological processes and identified cardiac/non-cardiac causes of breathlessness.	[87]

**Table 3:** Machine Learning Models in ECG for Cardiovascular Disease Diagnosis.

Research Direction	Validation Type	Model Type/Technology	Research Content (Diagnostic Target)	Data & Validation Status	Core Performance Metrics	Ref.
HFpEF Detection	Internal and External	Convolutional Neural Network (CNN)	Detect Heart Failure with Preserved Ejection Fraction (HFpEF) based on 12-lead ECG	Derivation cohort (including blinded test set) External validation cohort	Blinded test set of derivation cohort: AUROC = 0.92, Sensitivity = 0.98, Specificity = 0.63 External validation cohort: AUROC = 0.80, Sensitivity = 0.99, Specificity = 0.60, Positive Predictive Value (PPV) = 0.68, Negative Predictive Value (NPV) = 0.98	[18]
HFrEF Detection	Internal and External	Convolutional Neural Network (CNN)	Detect Heart Failure with Reduced Ejection Fraction (HFrEF) based on 12-lead ECG	Key correlated features: lateral/anterior wall leads, heart rate, QT interval, QRS duration, T-axis	Internal validation: AUROC = 0.913 External validation: AUROC = 0.961	[89]
LV Systolic Dysfunction Screening	Internal and External	Convolutional Neural Network (CNN)	Screen left ventricular (LV) systolic dysfunction using 12-lead ECG images (detect LV ejection fraction [LVEF] < 40%)	Internal validation cohort External validation cohort	Internal validation: AUROC = 0.91 External validation: AUROC = 0.88–0.95	[90]
Prediction of Multiple Cardiovascular Conditions (Including HF)	Internal	ResNet-based Deep Learning (DL) Model	Predict multiple cardiovascular conditions (including Heart Failure [HF]) based on 12-lead ECG	Comparison with XGBoost model	AUROC was approximately 5% higher than XGBoost for 8 cardiovascular conditions (specific AUROC for HF not mentioned)	[91]
Detection of Diastolic Dysfunction and Elevated Filling Pressure	Internal	AI-Enabled ECG Model	Identify echocardiographically confirmed diastolic dysfunction and elevated left ventricular filling pressure based on 12-lead ECG	Focus on diastolic dysfunction grades (≥Grade 1, ≥Grade 2, Grade 3) and elevated filling pressure	Detection of elevated filling pressure: AUROC = 0.911 Identification of diastolic dysfunction ≥Grade 1: AUROC = 0.847 Identification of diastolic dysfunction ≥Grade 2: AUROC = 0.911 Identification of diastolic dysfunction Grade 3: AUROC = 0.943	[92]
Analysis of Gender-Specific ECG Parameters in HFpEF Patients	Internal	AI + Multiple Machine Learning Models (Gradient Boosting Machine, k-Nearest Neighbors, Logistic Regression, Random Forest, Support Vector Machines)	Analyze gender-specific ECG parameters in HFpEF patients to distinguish between male and female HFpEF patients	Comparison of performance across different models; focus on key influencing parameters of the Random Forest model	Random Forest model: Average accuracy = 84.7% (Key distinguishing parameters: smoking status, P-wave dispersion, P-wave amplitude, T-end P/(PQ × Age), Cornell product, P-wave duration)	[93]
HFpEF Detection	Internal and External	Interpretable Deep Learning Model (Ensemble Neural Network)	Early detection of HFpEF using 12-lead, 6-lead, and single-lead ECG	Enrolled 34,103 patients with normal left ventricular systolic function (ejection fraction ≥ 50%) from two hospitals; Internal validation (1979 cases), External validation (11,955 cases)	12-lead ECG model: Internal validation AUROC = 0.866, External validation AUROC = 0.869; High-risk group HFpEF development probability 33.6% vs. low-risk group 8.4% (*p* < 0.001); Model focused on QRS complex and T-wave	[94]

**Table 4:** AI Applications in CMR for Assessing HFpEF and Related Cardiovascular Diseases.

Research Direction	Validation Type	Model/Technical Framework	Research Content	Data & Technical Details	Core Performance Metrics	Ref.
HF Classification (Including HFpEF-Related Assessment)	Internal	4-Dimensional Self-Supervised Learning Framework	Classify heart failure (HF) using cine CMR (cine cardiac magnetic resonance) by integrating spatial and temporal information to improve diagnostic accuracy	The model is built using 3D spatial information and temporal dimension information from cine CMR, aiding in the selection of personalized treatment plans	AUROC = 0.8794 Accuracy = 0.8402	[95]
Prediction of CMR Diagnostic Results and Left Ventricular End-Diastolic Pressure (LVEDP)	Internal and Prospective	AI-Enhanced Non-Invasive CMR Assessment Method	Develop a non-invasive AI model to predict patients’ CMR diagnostic results and LVEDP (a key indicator for HFpEF assessment) using CMR data	The model is designed with a focus on clinical practicality and can be integrated into routine clinical workflows	Enhancing the information value of CMR, facilitating disease classification and diastolic function prediction	[96]
Screening and Diagnosis of 11 Types of Cardiovascular Diseases (CVDs)	Internal and External	Deep Learning-Based Two-Stage CMR Interpretation Paradigm (Backbone Network: Video-based Swin Transformer, VST)	Two-stage workflow: 1. Stage 1: Non-invasive screening for cardiovascular abnormalities using cine MRI 2. Stage 2: Disease diagnosis by combining cine MRI with late gadolinium enhancement (LGE) MRI	Covers 11 types of CVDs; validated on internal and external datasets; capable of identifying CMR features that are difficult for humans to detect	Screening model: AUROC = 0.988 ± 0.3% Diagnostic model: AUROC = 0.991 ± 0.0% The diagnostic model outperforms cardiologists in diagnosing pulmonary arterial hypertension (PAH)	[97]

**Table 5:** Applications of AI-Integrated Wearable Technologies in Cardiovascular Disease Detection and Remote Monitoring.

Research Direction	Validation Type	Model/Technology Type	Research Content	Data & Technical Details	Core Performance Metrics	Ref.
Detection of Left Ventricular Systolic Dysfunction (LVSD) via Single-Lead Electrocardiogram (ECG)	Internal	Noise-Adapted AI Model, Standard AI Model	Develop and validate models for detecting LVSD using noisy single-lead ECGs acquired from portable/wearable devices, with a focus on improving performance in noisy environments	Data: 385,601 paired ECG-transthoracic echocardiogram (TTE) samples (involving 116,210 patients) Models: A standard model and a noise-adapted model were developed; performance was compared under different Signal-to-Noise Ratios (SNR)	Noise-free test set: Both models achieved an AUROC of 0.90 Noisy test set (portable device noise, SNR = 0.5): The noise-adapted model reached an AUROC of 0.87, significantly outperforming the standard model (AUROC = 0.72) Subgroup performance: Consistent performance across subgroups stratified by age, sex, and ethnicity	[99]
Detection of LVSD via Single-Lead ECG from Apple Watch	Prospective	AI Algorithm + Apple Watch Single-Lead ECG	Validate the feasibility of detecting LVSD using single-lead ECGs from Apple Watches combined with an AI algorithm	Prospective study; two AI calculation strategies were adopted: “mean prediction within 30 days” and “ECG prediction closest in time to TTE”	“Mean prediction within 30 days” strategy: AUROC = 0.885 “ECG prediction closest in time to TTE” strategy: AUROC = 0.881	[100]
Differentiation of Heart Failure States (Compensated vs. Decompensated)	Internal	Machine Learning (ML)-Assisted Wearable Technology	Differentiate between compensated and decompensated HF states using seismocardiogram signals via ML-assisted wearable technology, exploring the potential for remote monitoring of HFpEF patients	Seismocardiogram signals collected by wearable devices were used, focusing on distinguishing between different pathological states of HF patients	Ability to differentiate compensated and decompensated HF states, with potential for remote monitoring of HFpEF patients	[13]
Direct Monitoring of Pulmonary Artery Pressure (PAP) for Heart Failure (HF) Hospitalization Reduction	Prospective	CardioMEMS Direct Pulmonary Artery Pressure Monitoring System	Validated in the CHAMPION trial; guide treatment for NYHA Class III HF patients based on pulmonary artery pressure measurements to reduce HF hospitalization rate	Large-scale randomized trial; follow-up over 17 months; subgroups including ejection fraction > 40% and ischemic cardiomyopathy patients	6-month HF hospitalization rate reduced from 0.44 (control) to 0.32 (treatment, 28% relative risk reduction); over 17 months, relative risk of hospitalization reduced to 37%; excellent safety (extremely low device-related complication rate, zero pressure sensor failures)	[101]

## Data Availability

No new data were created or analyzed in this study.
